# Detection of *Theileria luwenshuni* in sheep from Great Britain

**DOI:** 10.1186/s13071-016-1486-5

**Published:** 2016-04-13

**Authors:** L. Paul Phipps, Luis M. Hernández-Triana, Hooman Goharriz, David Welchman, Nicholas Johnson

**Affiliations:** Wildlife Zoonoses and Vector-Borne Disease Research Group, Animal and Plant Health Agency, Woodham Lane, Addlestone, Surrey KT15 3NB UK; Surveillance Intelligence Unit, APHA Winchester, Itchen Abbas, Winchester, SO21 1BX UK

**Keywords:** *Theileria luwenshuni*, Sheep, Great Britain, *Haemaphysalis punctata*

## Abstract

**Background:**

*Theileria* spp. are tick-borne protozoan parasites of the Phylum Apicomplexa, Order Piroplasmida that infect a wide range of wild and domestic animals. In Great Britain, *Theileria* spp. have been reported from livestock associated with transmission by the tick *Haemaphysalis punctata*. However, these reports have not been associated with disease. This study has investigated the cause of a disease outbreak accompanied by mortality in a flock of sheep grazing reclaimed marshland in north Kent.

**Findings:**

A polymerase chain reaction-reverse line blot assay indicated the presence of *Theileria* spp. in blood samples from five animals. Subsequent testing with a pan-piroplasm PCR of a larger panel of blood samples detected a piroplasm amplicon in 19 of 21 sheep submitted from the affected flock. Automated sequencing confirmed that these amplicons shared 99–100 % identity with *T. luwenshuni*.

**Conclusions:**

The clinical and PCR data suggest infection with *T. luwenshuni* was associated with disease and mortality in this flock.

**Electronic supplementary material:**

The online version of this article (doi:10.1186/s13071-016-1486-5) contains supplementary material, which is available to authorized users.

## Findings

*Theileria* Species of *Theileria* are tick-borne, protozoan blood parasites of the Phylum Apicomplexa, Order Piroplasmida and along with the species of the closely related genus *Babesia*, may infect wild and domestic animals worldwide [1]. *Theileria* spp. infections are transmitted through the bite of infected ixodid ticks and are characterised by the formation of schizonts within the cytoplasm of host lymphocytes where asexual division takes place to form merozoites, which subsequently infect circulating erythrocytes to form piroplasms. Sexual reproduction occurs in the gut of the tick vector resulting in motile kinetes which invade the tick salivary gland where further division occurs in sporoblasts to form infective sporozoites [2]. *Theileria parva* and *T. annulata*, the causative agents of East Coast fever and Tropical theileriosis, respectively, are important pathogens of cattle in tropical and sub-tropical regions of the Old World [3]. *Theileria equi* causes equine piroplasmosis and has a worldwide distribution in tropical and sub-tropical regions [4]. In small ruminants clinical disease has been historically associated with *T. lestoquardi* infection of sheep and described as malignant ovine theileriosis in the Mediterranean basin, north Africa and Asia [5]. Infection of goats with *T. lestoquardi* is reportedly benign. Recently, two new *Theileria* spp. causing clinical disease in sheep, *T. uilenbergi* and *T. luwenshuni*, have been described from northern China and associated with transmission by the hard tick *Haemaphysalis qinghaiensis* [6]. In a study to determine the prevalence and genetic diversity of piroplasms in northern Spain, three *Theileria* genotypes were identified in apparently healthy sheep, sharing 96.7–97.0 % similarity between their 18S rRNA gene sequences: *T. ovis*, *T.* sp. OT1 (99.6 % similarity with the recently described pathogenic piroplasm *Theileria* sp. China 1), and *Theileria* sp. OT3 [7]. In a more recent study, using a multiplex DNA bead-based suspension array (Luminex xMAP) to detect piroplasms in sheep from northern Spain, the authors detected five different piroplasms including *Theileria *sp. OT3 in 34.8 % of the samples, *T. ovis* in 20.9 %, and at lower prevalences *Babesia motasi* (12.3 %), *T. luwenshuni*/OT1 (10.5 %) and *Babesia ovis* (6.3 %) [8]. During the two year period of this study, more than 10,000 questing ticks of all life-cycle stages were captured in the field and eight species of ticks identified including; *Ixodes ricinus*, *H. punctata*, *Dermacentor reticulatus* and *H. inermis* and sporadic captures of *D. marginatus*, *I. frontalis*, *Rhipicephalus bursa* and *H. sulcata*. The authors suggest a relationship between *T. luwenshuni*/OT1 and a *Haemaphysalis* sp. in the study area.

In Great Britain theileriosis has been reported from South and North Wales associated with transmission by *H. punctata*. Lewis and co-workers [9] isolated a piroplasm described as *T. ovis* when they inoculated a splenectomised sheep with a pooled blood sample taken from 50 healthy ewes grazing common land in coastal South Wales, where *H. punctata* was the only prevalent tick species. However, in North Wales, a morphologically similar piroplasm described as *T. recondita* was isolated by transmission via field-caught female *H. punctata* to splenectomised sheep [10]. The authors reported pyrexia with body temperatures rising to a maximum 41.5 °C during peak phase of parasitaemia followed by recovery.

During April 2005 mortality associated with very heavy infestations by the tick *H. punctata*, was investigated in a group of 60 ewes and their lambs grazing north Kent marshland. More than 25 ewes died in the group which was part of a 900 ewe flock. However, it was observed that only the animals grazing one pasture where ticks were prevalent were affected. Post-mortem examination of an affected ewe revealed a very heavy burden of *H. punctata,* especially on the underside of the animal. The lips and tongue were swollen and oedematous, and there was also oedema of the conjunctivae and subcutaneous oedema around the eyes, and elsewhere over the face and throat. There were petechial haemorrhages over the oral mucosa and extensive subcutaneous congestion over the underside of the animal. The lungs were oedematous and mottled and excessive froth was present in the trachea and bronchi. The spleen was enlarged, the kidneys pale and the blood was watery.

Blood samples were taken by venepuncture into EDTA sample tubes from 2, 2-week old lambs and twelve random ewes in the same field. Giemsa staining of methanol fixed, thin blood smears was conducted using standard procedures [11]. This revealed the presence of anaplasmoid inclusions in the red cells of both of the lambs and ten of the ewes, accompanied by a moderate to severe macrocytic, hypochromic anaemia in the lambs and six of the ewes and a neutrophilia in the lambs and seven of the ewes. Further tests on blood samples from the sheep using PCR and reverse line blot (RLB) [12] at the University of Utrecht demonstrated the presence of a *Babesia* sp., a *Theileria* sp. and an *Anaplasma / Ehrlichia* sp. other than *A. marginale, A. centrale, A. ovis* and *A. phagocytophilum*. However, the University was unable to offer further specific molecular tests at the time. During October 2012 a further 21 EDTA blood samples were taken from yearling sheep grazing the same pasture and subjected to a pan piroplasm PCR. Total DNA was extracted from 200 μl whole blood using the DNAeasy Blood and Tissue Kit following the manufacturer’s spin-column protocol (QIAgen, Manchester, UK).

Pan Piroplasm PCR was conducted using the primers PIRO-A (5’-AATACCCAATCCTGACACAGGG-3’) and PIRO-B (5’-TTAAATACGAATGCCCCCCAAC-3’) [13] that gives a 423 base pair (bp) amplicon derived from the 18S rRNA gene. Sample DNA was added to a mixture of primers and SYBR® Green JumpStart™ Taq ready mix (Sigma-Aldrich, St Louis, USA) in a total volume of 40 μl. Amplification was achieved with the following conditions: 94 °C for 10 s (s), followed by 45 cycles of 94 °C for 30 s, 58 °C for 30s and 72 °C for 1 min. Samples were separated on a 1 % agarose gel containing SYBR safe (Sigma) and visualised by ultraviolet illumination. Samples were sequenced using the primers PIRO-A and PIRO-B with the ABI PRISM® BigDye® Terminator v3.1 Cycle Sequencing Kit (Applied Biosystems, Life Technologies Ltd., Paisley UK) following the manufacturer’s instructions. Neighbour-joining analysis was performed using MEGA v.5 software [14].

**Fig. 1:**
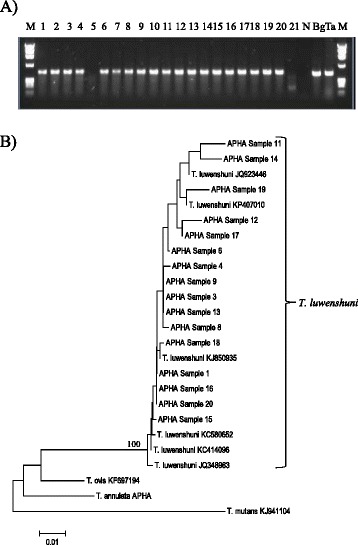
**a** Gel analysis of pan-piro PCR on blood samples from Cooling Marsh sheep. Sheep samples are in tracks 1–21. M is DNA marker (fX154), N is a no-template negative control, Bg is *B. gibsoni* positive control and Ta is *T. annulata* positive control. **b** Neighbour Joining tree constructed from sequences (407 bp) of the 18S rRNA gene including samples from infected ewes from Kent and representative *Theileria* sp. of ruminants. Only bootstrap values higher than 70 % are shown in the tree below. Details of sequences included in the phylogeny are provided in Additional file 1: Table S1

Nineteen samples produced a clear amplicon of a size corresponding with piroplasm controls (Fig. 1a). Each amplicon was sequenced using flanking primers. Sixteen of the 21 samples produced sequence suitable for further analysis. A representative sequence has been submitted to GenBank (Accession number KU234526). BLAST searches on each sequence indicated that they all shared 100 % identity with *T. luwenshuni* (GenBank sequence: KP407010) and 99.8 % identity with *Theileria* sp. OT1 (GenBank sequence: AY533143). Neighbour Joining (NJ) analysis of a subset of the Kent *Theileria* sequences compared with a range of ruminant *Theileria* spp. demonstrates the close association with *T. luwenshuni* with 100 % bootstrap values (see Fig. 1b). The dataset was also analysed using the Maximum Likelihood method using bootstrap values of 1000 replications to assess phylogenetic relationships (tree not shown). We obtained a similar topology as the NJ tree with the group of *T. luwenshuni* supported with 100 % bootstrap values.

Prior to the application of molecular phylogenetics to the delineation of blood parasites, *Babesia motasi, T. recondita* and *Ehrlichia phagocytophila* (now named *Anaplasma phagocytophilum*) were recognised tick-borne haemoparasites of sheep in the United Kingdom (UK). Field surveys of the site detected the likely vector of *T. luwenshuni*, *H. punctata*, but failed to detect *T. luwenshuni* in a group of adults and nymphs. However, an amplicon of the correct size was detected in DNA extracted from one nymph and sequencing obtained 392 base pair sequence (GenBank Accession number KU234527). BLAST analysis, as described above, demonstrated that this amplicon shared 99 % identity with *B. motasi* 18S ribosomal subunit gene. This species is a relatively benign *Babesia* sp. previously described by Lewis and Herbert (1980) [15] from sheep in North Wales. In field conditions, *H. punctata* larvae feed on small mammals and birds and rarely feed on sheep [10]. It appears that transmission of *T. luwenshuni* is typical of the genus. The parasite is first picked up from infected carrier animals by the nymphal stage, which readily feeds on sheep, and passed on to the subsequent adult feeding stage i.e. transtadial transmission. In contrast, *Babesia* sp. transmission is transovarial. Female hard tick species acquire *Babesia* infection whilst feeding on infected carrier animals and pass the infection on to next generation larvae via the eggs. The subsequent life-cycle stages retain infection through feeding and metamorphosis and may transmit infection through to the first generation adult stage at least. This provides explanation for the detection of *B. motasi* in our field-caught *H. punctata* nymph. From the original RLB work carried out on this disease incident, it appears that an *Ehrlichia* / *Anaplasma* species may also subsequently be identified which has not been recorded in the UK until this time.

## Conclusions

A pan-piroplasm PCR and subsequent sequencing of the amplicons suggests that the flock of sheep investigated in this study is infected with *T. luwenshuni* and that this could be the causative agent for disease and deaths observed on this farm in 2005. Clinical signs of acute babesiosis include fever, anaemia, icterus, haemogobinuria [16] whereas signs associated with acute or fatal theileriosis also include purulent nasal discharge, pneumonia, ataxia, dyspnoea in fatal *T. orientalis* infection in Asian water buffalos [17], fever, anaemia, serous nasal discharge, swelling of eyelids, ears and jowls, and pneumonia including frothy discharge from nostrils in the case of East Coast fever, caused by *T. parva* ([18]). The post mortem findings in the ewe and the moderate to severe anaemia in both the ewes and lambs were consistent with the reported effects of pathogenic *Theileria* spp. [19]. We conclude that *T. luwenshuni* has been indigenous to the UK and that previous investigations of cryptic ovine *Theileria* infection in Great Britain [9, 10] may be due to this species. Alternatively, *T. luwenshuni* could have been introduced from China where it is increasingly prevalent [20]. One potential source could have been the introduction of Asian deer species such as Sika (*Cervus nippon*), muntjac (*Muntiacus reevesi*) and Chinese water deer (*Hydropotes inermis*) over the past 150 years.

There has been no significant disease outbreak in the sheep on these marshes since 2005. The mortality in 2005 coincided with very heavy tick infestations, possibly triggered by the prevailing weather conditions. The ewes on this land were part of a closed flock and only rams were brought in for breeding purposes. This outbreak is consistent with an introduction of *T. luwenshuni* during 2004 via infected *H. punctata* nymphs on replacement rams followed by transmission by the subsequent adult population during the spring of 2005 causing clinical disease in a naïve flock of ewes and lambs. In following years the development of a stable endemicity [21] and subsequent low level of clinical disease may have been promoted through regular tick challenge of carrier ewes and protection of lambs by colostrum derived antibody, as suggested by the high infection rate of yearling sheep in this study. It is also feasible that heavy tick infestations may immunosuppress their hosts either via general debilitation and the effects of tick salivary proteins or via infection with known immunosuppressive tick-borne pathogens such as *Anaplasma* sp. thus promoting fulminating *Theileria* infection.

## Additional file

Additional file 1: Table S1. Sample details of sequences used in the phylogeny of *T. luwen* *shuni* in the United Kingdom. (DOC 33 kb)
