# Supplementary material for: Detection of Theileria luwenshuni in sheep from Great Britain
Source: Parasit Vectors. 2016 Apr 13;9:203. doi: 10.1186/s13071-016-1486-5 (PMC4831081; doi:10.1186/s13071-016-1486-5)
Supplement: Additional file 1: Table S1. — Sample details of sequences used in the phylogeny of T. luwen shuni in the United Kingdom. (DOC 33 kb) [file 13071_2016_1486_MOESM1_ESM.doc]

**Supplementary Table S1: Sample details of sequences used in the phylogeny of *T. luwenshuni* in the United Kingdom.**

| **GenBank Accession Number** | **Species** | **Country of origin** | **Source of sample** |
| --- | --- | --- | --- |
| JQ923446 | *T. luwenshuni* | China | Sheep |
| KP407010 | *T. luwenshuni* | China | Red deer |
| KJ850935 | *T. luwenshuni* | China | Sheep |
| KC580652 | *T. luwenshuni* | China | *Haemaphysalis longicornis* |
| KC414096 | *T. luwenshuni* | China | Sheep |
| JQ348963 | *T. luwenshuni* | China | Goat |
| KF697194 | *T. ovis* | Iran | Sheep |
| KJ941104 | *T. mutans* | Ethiopia | *Amblyomma variegatum* |
